# A hydrophobic gelatin fiber sheet promotes secretion of endogenous vascular endothelial growth factor and stimulates angiogenesis†

**DOI:** 10.1039/d0ra03593a

**Published:** 2020-06-30

**Authors:** Yosuke Mizuno, Tetsushi Taguchi

**Affiliations:** Graduate School of Science and Technology, University of Tsukuba 1-1-1 Tennodai Tsukuba Ibaraki 305-8577 Japan; Polymers and Biomaterials Field, Research Center for Functional Materials, National Institute for Materials Science 1-1 Namiki Tsukuba Ibaraki 305-0044 Japan TAGUCHI.Tetsushi@nims.go.jp +81-29-860-4498

## Abstract

In tissue engineering and regenerative medicine, the formation of vascular beds is an effective method to supply oxygen and nutrients to implanted cells or tissues to improve their survival and promote normal cellular functions. Various types of angiogenic materials have been developed by incorporating growth factors, such as vascular endothelial growth factor, in biocompatible materials. However, these exogenous growth factors suffer from instability and inactivation under physiological conditions. In this study, we designed a novel angiogenic electrospun fiber sheet (C16-FS) composed of Alaska pollock-derived gelatin (ApGltn) modified with hexadecyl (C16) groups to induce localized and sustained angiogenesis without growth factors. C16-FS was thermally crosslinked to enhance its stability. We demonstrated that C16-FS swells in phosphate-buffered saline for over 24 h and resists degradation. Laser doppler perfusion imaging showed that C16-FS induced increased blood perfusion when implanted subcutaneously in rats compared with unmodified ApGltn-fiber sheets (Org-FS) and the sham control. Furthermore, angiogenesis was sustained for up to 7 days following implantation. Immunohistochemical studies revealed elevated nuclear factor-κB and CD31 levels around the C16-FS implantation site compared with the Org-FS implantation site and the control incision site. These results demonstrate that C16-FS is a promising angiogenic material to promote the formation of vascular beds for cell and tissue transplantation without the need for growth factors.

## Abstract 2

*In vivo* long-term growth factor-free angiogenesis by LPS-mimicking C16-modified gelatin based electrospun fiber sheet.

## Graphical abstract

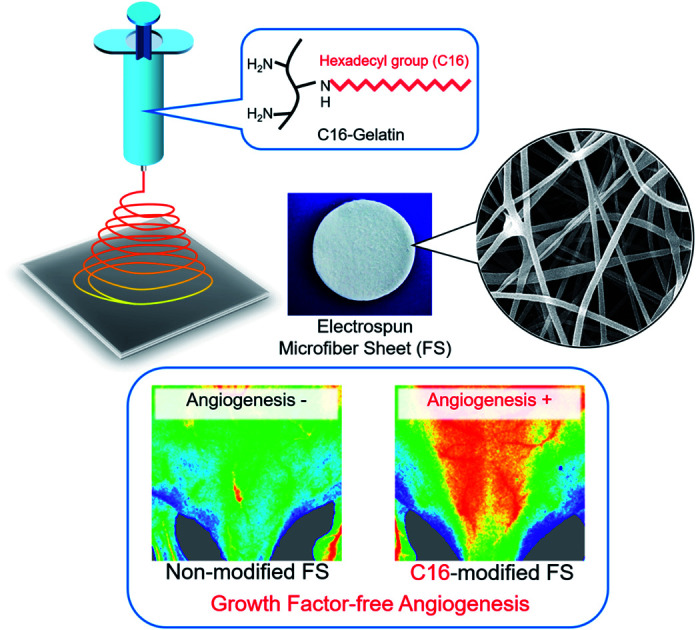

## Introduction

Tissue engineering and regenerative medicine is a promising approach to repair or replace damaged tissues and organs. Clinical studies in this field are mainly based on injecting cell suspensions or transplanting engineered tissues at a target site, where they are expected to serve as a substitute for the damaged tissue. However, the survivability of implanted cells and tissues is low because the supply of oxygen and nutrients *via* native blood vessels is insufficient.^1,2^ To overcome these challenges, vascular beds can be used to enhance the survivability and function of implanted cells or tissues.^3–5^

Biomaterials researchers have developed various angiogenic materials by incorporating growth factors (GFs), such as vascular endothelial growth factor (VEGF), basic fibroblast growth factor (bFGF), and stromal cell-derived factor (SDF)-1, in hydrogels,^6–8^ particles,^9,10^ and porous scaffolds.^11,12^ The sustained release of GFs from these materials promotes angiogenesis *in vitro* and *in vivo* as well as wound healing,^13–15^ regeneration in an infarcted heart,^16^ and survivability of implanted islets.^17^ However, GFs are easily inactivated under physiological conditions, compromising their bioactivity.^18–21^

On the other hand, Kanno *et al.* reported that inoculation with *Pseudomonas aeruginosa*, a Gram-negative bacterium, promotes re-epithelialization and angiogenesis by attracting neutrophils to the wound site.^22^ However, the bacteria proliferate rapidly, leading to an unstoppable cytokine storm and sepsis. These inflammatory response and angiogenesis are caused by lipopolysaccharide (LPS), a constituent of the cell wall of Gram-negative bacteria. Besides, saturated fatty acids (SFAs), a partial structure of LPS, promote the secretion of inflammatory cytokines and growth factors from inflammatory cells *via* the Toll-like receptor (TLR) 4-mediated pathway.^23–28^ We previously reported that SFA-modified Alaska-pollock-derived gelatin (ApGltn), self-assembles into a hydrogel when hydrated with phosphate-buffered saline (PBS) and stimulated VEGF secretion and angiogenesis *in vitro* and *in vivo*.^29^ However, the SFA-ApGltn hydrogels could not maintain their shapes under physiological conditions as they are vulnerable to enzymatic degradation. Chemical,^30,31^ enzymatic,^32^ and physical^33,34^ methods have been used to make gelatin-based materials resistant to enzymatic degradation *in vivo*.^35^ However, in the case of electrospinning, it is difficult to attain homogenous crosslinking and remove residual crosslinker and byproducts with chemical and enzymatic methods. Therefore, we utilized a physical method, thermal crosslinking, to fabricate electrospun microfiber sheets because of its simplicity and the ability to control the crosslinking density by changing the heating time.^34,36–38^

In the present study, we used a hexadecyl group (C16) as an immune activator^25^ to synthesize C16-modified ApGltn (C16-ApGltn), then used electrospinning to fabricated C16-ApGltn-based fiber sheets (C16-FSs) then thermally crosslinked to increase the crosslinking density and enhance their stability. The physicochemical properties of the C16-FSs with different crosslinking densities were evaluated in terms of the water contact angle, swelling ratio, and degradation rate. Moreover, the *in vivo* angiogenic properties of the C16-FSs in rats were quantified by laser doppler perfusion imaging (LDPI) and immunohistochemical staining.

## Experimental

### Materials

ApGltn (molecular weight = 33 000 g mol^−1^) was purchased by Nitta Gelatin (Osaka, Japan). Hexadecanal was purchased from Tokyo Chemical Industry (Tokyo, Japan). 2-Picoline borane (pic-BH_3_) was purchased from Junsei Chemical (Tokyo, Japan). Collagenase and Dulbecco's PBS were from Nacalai Tesque (Kyoto, Japan). 2,4,6-Trinitrobenzensulfonic acid (TNBS), triethylamine, 2-aminoethanol, ethanol, dimethyl sulfoxide (DMSO), and 10% formalin neutral buffer solution were purchased from Wako Pure Chemical Industries (Osaka, Japan).

### Synthesis of C16-ApGltn

Before the modification ApGltn with hexadecyl groups, the amino group concentration in the gelatin was quantified by the TNBS method, as previously reported.^39–41^ Briefly, 100 μl of 0.1 w/v% ApGltn dissolved in 1 : 1 DMSO/H_2_O was dispensed into each well of a 48-well plate. 100 μl of a solution of 0.1% TNBS and 0.1% triethylamine in 1 : 1 DMSO/H_2_O was then added to each well. The plate was then shaken for 1 min using a microplate shaker and incubated or 2 h at 37 °C. Then, the absorbance at 335 nm in each well was measured with a microplate reader (Spark 10M; Tecan, Männedorf, Switzerland) to calculate the number of amino groups. 2-Aminoethanol (which has a known concentration of amino groups) was used instead of ApGltn to generate a calibration curve.

C16-ApGltn was synthesized by reductive amination^42–44^ as described previously^41^ (Fig. S1†). Briefly, 20 w/v% ApGltn was dispersed in a 30% ethanol aqueous solution and dissolved by heating at 50 °C. Hexadecanal and 2-picoline borane were dissolved in ethanol and added to the ApGltn solution in 1.5-fold molar excesses relative to the remaining amino groups in the ApGltn. The reaction was carried out for 18 h. The reaction solution was then added dropwise to cold ethanol (10 times the reaction solution volume) to precipitate the C16-ApGltn, which was then isolated by suction filtration using a glass filter. The C16-ApGltn was then re-dispersed in ethanol three times to remove remaining the hexadecanal and 2-picoline borane. The C16-ApGltn precipitate was finally vacuum-dried at 25 °C, yielding a white powder. Unmodified ApGltn (Org-ApGltn) was prepared by the same protocol used for C16-ApGltn without the addition of hexadecanal and 2-picoline borane. The modification ratio of hexadecyl groups in C16-ApGltn was quantified using the TNBS method and comparing the absorbance at 335 nm with that of Org-ApGltn. The degree of modification was further confirmed by ^1^H nuclear magnetic resonance (^1^H-NMR) (JNM-AL300; JEOL, Tokyo, Japan).

### Fabrication and characterizations of C16-FS

**Fig. 1:**
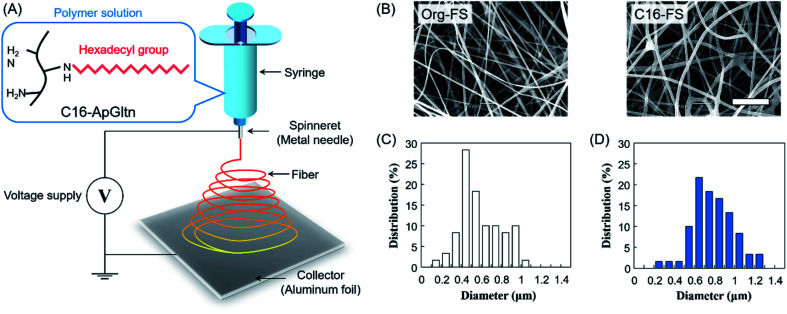
Fabrication and characterization of electrospun fiber sheets. (A) Electrospinning of C16-ApGltn into a fiber sheet (FS). (B) SEM images of FSs (scale bar = 10 μm). (C and D) Histograms of the fiber diameters in (C) Org-FS and (D) C16-FS, as quantified using Image J (*n* = 60).

Microfiber sheets were fabricated by electrospinning the gelatin solution. Briefly, Org-ApGltn was dissolved in a 40% aqueous ethanol solution at 30 w/v%, and C16-ApGltn was dissolved in a 50% EtOH aqueous solution at 15 w/v%. Electrospinning was conducted using a NANON-03 instrument (MECC, Japan) at room temperature (25 °C) (Fig. 1A). Org- and C16-ApGltn solutions were extruded from an 18 G needle at a feed rate of 1 ml h^−1^ with voltages of 20 and 25 kV, respectively. The obtained Org-FS and C16-FS were observed with a scanning electron microscope (SEM) (S-4800 ultrahigh-resolution SEM, HITACHI, Japan), and the fiber diameters were measured using Image J software (*n* = 60). Fourier transform infrared spectroscopy (FT-IR) (ALPHA II; Bruker Corp., Billerica, MA, USA) was conducted on a C16-FS to confirm the presence of hexadecyl groups. Fiber sheets were then thermally crosslinked in a vacuum oven at 150 °C for 4 h to improve their degradation resistance.

### Water contact angle measurements

To evaluate the hydrophobicity of the fiber sheets, water contact angle measurements were conducted using a DropMaster DM-701 (Kyowa Interface Science, Japan). Contact angle measurement was initiated 0.5 s after a 3 μl PBS droplet was dropped on the fiber sheet and continued for up to 15 s. The surface contact angle of the PBS droplet on the fiber sheet was then analyzed using FAMAS software.

### Swelling ratio

After 4 h of thermal crosslinking, fiber sheets were cut into 10 mm-diameter circles, each of which was weighed to determine the dry weight (*W*_d_). Fiber sheets were then immersed in PBS at 37 °C for up to 1440 min. The swollen fiber sheets were weighed at each time point to determine the swollen weight (*W*_s_). Hence, the swelling ratio of each sheet was calculated using the following equation:
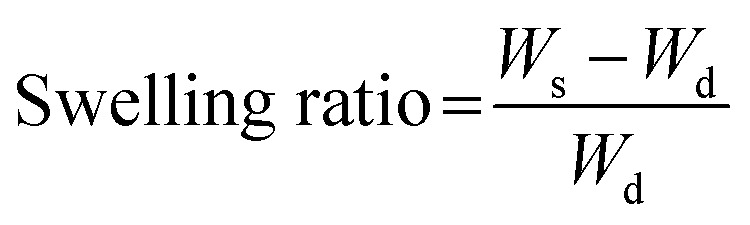


### Enzymatic degradation of C16-FS

The enzymatic degradation of Org-FS and C16-FS was evaluated using collagenase. Briefly, after 4 h of thermal crosslinking, Org-FS and C16-FS were cut into 10 mm-diameter circles and immersed in PBS at 37 °C for 1 h for pre-swelling. The swollen FSs were then immersed in a solution of 5 U ml^−1^ collagenase in PBS and incubated at 37 °C for up to 240 min. The FSs were weighed at each time point to determine the degradation profiles.

### Evaluation of angiogenesis in mice

Animal experiments were approved by the Animal Care and Use Committee of National Institute for Material Science (NIMS), Japan, and were performed in accordance with NIMS Regulations Pertaining to Animal Testing. To evaluate the angiogenic properties of C16-FS, the FSs were implanted subcutaneously in mice backs and evaluated by blood flow imaging and histological observation. Thermally crosslinked Org-FS and C16-FS were cut into 10 mm-diameter circles. A total of 24 hairless mice (Hos:HR-1, female, 4 weeks old; Hoshino Laboratory Animals, Japan) were anesthetized by isoflurane inhalation. An incision was made in the back of each mouse, the FS was implanted, and the incision was sutured closed. Sham surgery was also conducted by making an incision and then sutured without implanting FSs. The FSs were implanted near the mice's tail roots to avoid the blood flow instability caused by the movement associated with breathing.

The blood flows around the implant site was measured using an LDPI system (OZ-2, OMEGAWAVE, Japan) immediately after and 1, 2, 3, 7, 14, and 24 days after the implantation. To stabilize and control the blood flow rate in the mice for imaging, the isoflurane concentration was adjusted until each mouse's breathing rate was 1 breath per s. To analyze the mechanisms of angiogenesis *in vivo*, immunohistochemical staining was conducted on different mice from those used for the LDPI experiment. 3 days after the subcutaneous implantation of Org-FS and C16-FS, the mice were sacrificed using somnopentyl. The FSs and the surrounding tissue were dissected and fixed in a 10% formaldehyde neutral buffer solution. Tissue sections were stained with hematoxylin and eosin (H&E); nuclear factor-κB (NF-κB), VEGF, CD31, and myeloperoxidase (MPO) were stained as markers of inflammation and angiogenesis. Stained areas of NF-κB, VEGF, and CD31 were quantified using Image J.

### Statistical analysis

Data are shown as mean ± standard deviation (SD). Differences between groups were evaluated for statistical significance with the Student's *t*-test. Differences were considered statistically significant when *p* < 0.05.

## Results and discussion

### Synthesis of C16-ApGltn

C16-ApGltn was synthesized by reacting the residual amino groups in ApGltn with hexadecanal followed by reduction with 2-picoline borane. The yields of Org- and C16-ApGltn were 90.2% and 82.0%, respectively (Table 1). The modification ratio of C16-ApGltn was 24.0 mol%, as measured by the TNBS method. The ^1^H-NMR spectra shown in Fig. S2† show peaks at 1.4 ppm in the C16-ApGltn spectrum, which correlates with a strong peak at the same chemical shift in the hexadecanal spectrum, confirming the substitution of hexadecyl groups into ApGltn.

**Table 1:** Characterization of Org-FS and C16-FS

Abbreviations	Sheet thickness (μm)	Fiber diameter (μm)
Org-FS	223 ± 17	0.58 ± 0.20
C16-FS	276 ± 26	0.78 ± 0.20

### FS fabrication

The FT-IR spectra of the FSs (Fig. S3†) show that the absorbance of C–H at 2853 and 2927 cm^−1^ was increased in the C16-FS spectrum compared with the Org-FS spectrum, which was attributed to the modification of the alkyl chains with hexadecyl groups in C16-ApGltn. Therefore, C16-ApGltn molecules but C16-FS were expected to be more hydrophobic than Org-ApGltn and Org-FS. Org-FS and C16-FS were fabricated by accumulating electrospun fibers into non-woven sheets. The mean thicknesses of Org-FS and C16-FS were 223 ± 17 μm and 276 ± 26 μm, respectively (Table 1). The SEM images of Org-FS and C16-FS shown in Fig. 1B show that the fibers were successfully formed and molded into a non-woven fabric-like structure. The mean fiber diameter of C16-FS (0.78 ± 0.20 μm) was larger than that of Org-FS (0.58 ± 0.20 μm), as measured from the SEM images. Histograms of the fiber diameters are shown in Fig. 1C and D; these data show that over 70% of the fibers in the Org-FS were between 0.3 and 0.8 μm, whereas the same percent in the C16-FS were between 0.5 and 1.0 μm. After the thermal crosslinking, fiber diameters of Org- and C16-FS were 0.61 ± 0.13 μm and 0.72 ± 0.29 μm, respectively, which were not significantly different with that before crosslinking. Besides, fiber morphologies and histograms of Org- and C16-FS were quite similar to that before crosslinking (Fig. S4†). ApGltn has significantly lower sol–gel transition temperature (21.2 °C) than porcine and bovine-derived gelatin (31.2 °C) due to the lower content of proline and hydroxyproline, which contribute to forming the helical structure of gelatin.^45,46^ Therefore, ApGltn in FSs was considered to have a random coil and amorphous structure because electrospinning was conducted at room temperature (25 °C).^47^ The previous study reported that uncrosslinked and thermally-crosslinked gelatin fibers demonstrated similar dependence on elastic modulus and tan *δ*, which suggested that the thermally-crosslinked FSs retained amorphous structure.^48^

### FS hydrophobicity

**Fig. 2:**
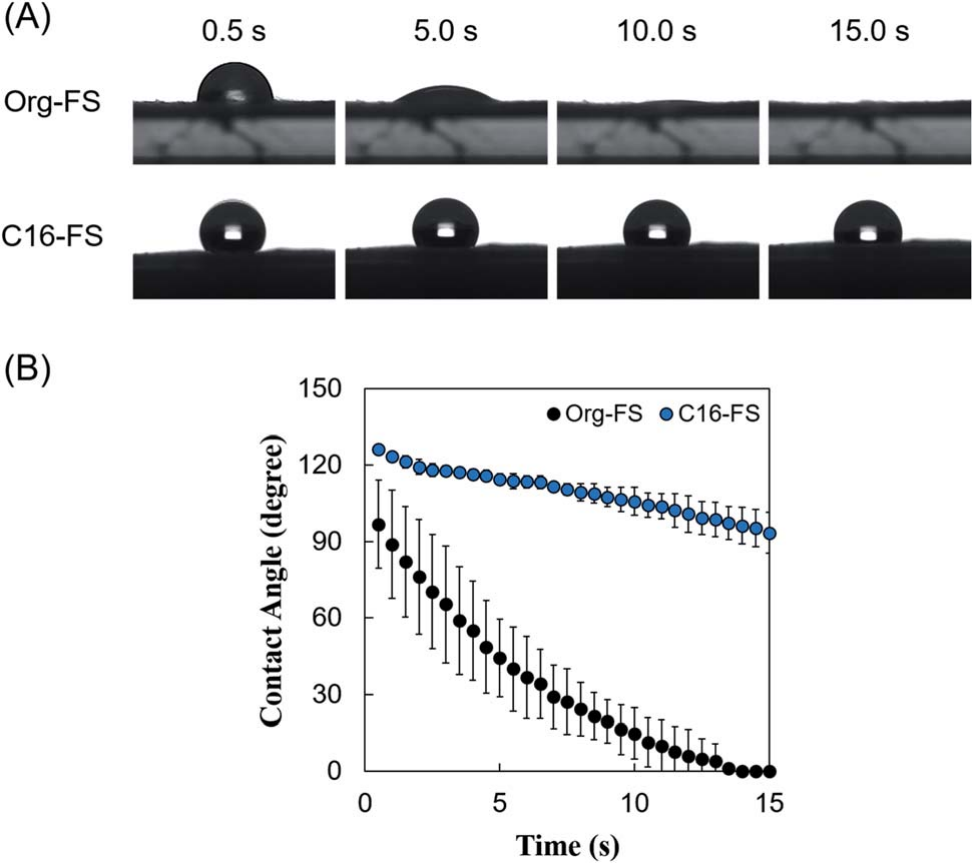
Surface contact angle measurements of Org-FS and C16-FS. (A) Representative images of the behavior of PBS droplets over 15 s on Org-FS and C16-FS after 4 h of thermal crosslinking. (B) Quantification of the decrease in the contact angle over time. Error bars represent SDs of each point (*n* = 3).

The surface contact angle on each FS was measured to evaluate the surface hydrophobicity, which may affect the water absorption rate. Fig. 2A shows the behavior of PBS droplets on Org-FS and C16-FS. 0.5 s after dropping the PBS droplets, the surface contact angle of that on Org-FS was already smaller than that on C16-FS. The PBS droplets were then quickly absorbed into the Org-FS compared with the C16-FS. Fig. 2B shows the rates of decrease in the contact angles of PBS droplets on Org-FS and C16-FS over 15 s. The surface contact angle was consistently higher on C16-FS than on Org-FS over 15 s because the modification of hexadecyl groups in C16-ApGltn resulted in higher hydrophobicity. Moreover, the hexadecyl groups in C16-ApGltn were considered to be localized on the surface of the C16-FS because the FSs were fabricated in air, and hydrophobic molecules tend to move toward air–water interfaces.^49^ We previously confirmed that hydrophobic groups conjugated to gelatin molecules tended to localize on the surfaces of thermally crosslinked microparticles by X-ray photoelectron spectroscopy.^50^ Owing to this surface hydrophobicity, the C16-FS repelled PBS droplets and delayed their absorption into the FS.

### Swelling ratio

**Fig. 3:**
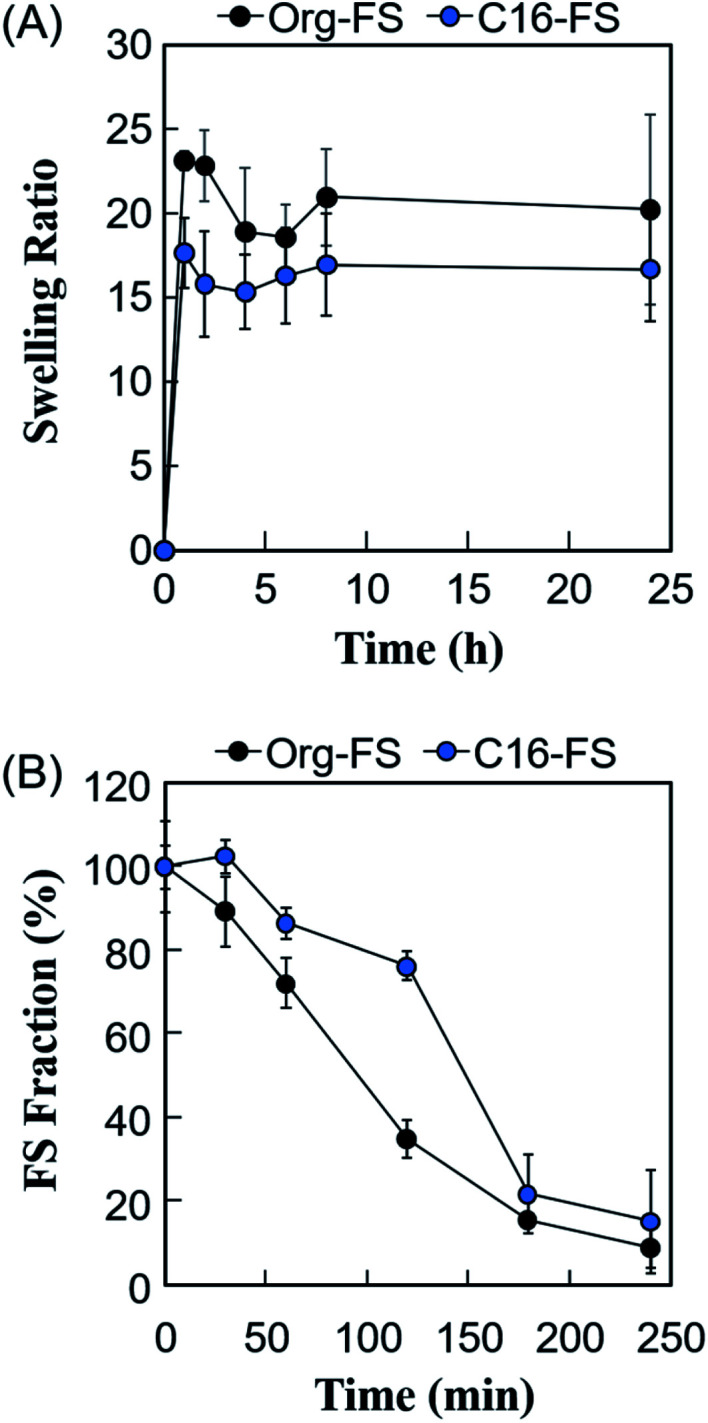
(A) Swelling ratios of Org-FS and C16-FS in PBS at 37 °C (*n* = 5). (B) Enzymatic degradation rates of Org-FS and C16-FS. Error bars represent SDs of each point (*n* = 4).

The swelling ratios of the FSs were measured to evaluate the effect of the hexadecyl groups in C16-FS on its stability in water (Fig. 3A). After immersing the FSs in PBS, they quickly absorbed PBS, as evidenced by significant weight gain in the 1 h of swelling. Both FSs then continued absorbing water and gaining weight over time, reaching steady states after 8 h. Although the swelling ratio of C16-FS was significantly lower than that of Org-FS for the first 2 h of swelling, no significant difference between them was observed after 4 h until 24 h. The swelling ratio basically depends on the crosslinking density in a polymer network; higher crosslinking densities result in less swelling, while lower crosslinking densities lead to increased swelling. However, the crosslinking density of C16-FS was considered to be lower than that of Org-FS due to the substitution of hexadecyl groups for amino groups, which contributes to amide bonding *via* dehydration condensation; this should lead to a higher swelling ratio in C16-FS. Therefore, the relatively swelling ratio of C16-FS despite its lower crosslinking density was attributed to physical crosslinking between hexadecyl groups (C16) in the C16-ApGltn molecules. We previously reported that the modification of hydrophobic groups in gelatin molecules contributes to decreased swelling ratios and increased mechanical strength in hydrogels, which is consistent with the results of the present study.^39^

### Enzymatic degradation of FSs

The effect of the hexadecyl groups in C16-FS on degradation rate was evaluated by applying collagenase, an enzyme that cleaves peptide bonds in gelatin and collagen, and measuring the weight over time. Before the degradation test, Org-FS and C16- FSs were pre-swelled in PBS for 1 h. Fig. 3B shows the degradation profiles of Org-FS and C16-FS in a collagenase solution. Results show that the weights of these FSs gradually decreased with time, and the weight of remaining C16-FS was higher than that of Org-FS after 120 min. These results were attributed to the suppressed swelling ratio of C16-FS for the first 2 h, which lead the delayed uptake of collagenase molecules into fibers. However, the weights of Org-FS and C16-FS were not significantly different after immersion in the collagenase solution for 180 min because both FSs broke into small fragments, leading to rapid degradation.

### Angiogenesis evaluation *in vivo*

**Fig. 4:**
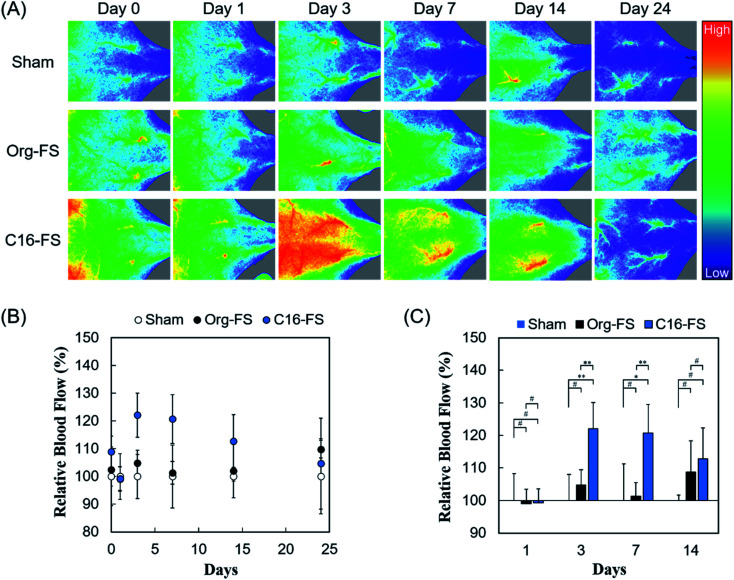
(A) LDPI images of mice backs after the sham implantation and implantation of Org-FS or C16-FS. The hues in images indicate the intensity of blood perfusion: red and blue indicate higher and lower perfusion, respectively. (B) Blood flow over 24 days, as quantified from the LDPI images. The blood flow values were normalized to that in the mice treated with the sham operation (control) for each day. Error bars represent SDs of each point (*n* = 4–5). (C) Comparison of mean blood flows in different groups on each day (* indicates *p* < 0.05, ** indicates *p* < 0.01, and ^#^ indicates *p* > 0.05).

The *in vivo* angiogenic properties of C16-FS were evaluated using LDPI and histological observations. Fig. 4A shows that the LDPI results after subcutaneous FSs implantation in mice, followed by quantification of blood flow at the implantation site. There were no significant differences between the LDPI images or blood flow in mice with Org-FS implanted, mice with C16-FS implanted, and control mice that received the sham operation (control) immediately after the implantation (Day 0), or on Day 1. However, on Day 3, the blood flow in the tissues around the C16-FS implantation site was increased, and visible blood vessels were observed, indicating an angiogenic response; however, the blood flow decreased after Day 3 until Day 24 (Fig. 4A). The quantitative analysis of blood flow over 24 days is shown in Fig. 4B. The blood flow measured on each day was normalized to that of the sham group on the corresponding; thus, the sham group was used as a reference and consistently exhibited 100% blood flow. There was no statistically significant difference in the mice with Org-FS implanted compared with the sham group over 24 days. However, in the C16-FS group, the blood flow was elevated (122%) on Day 3 and sustained until Day 7, after which time it gradually decreased to 104% on Day 24. The time-dependent decrease in blood flow around the C16-FS was attributed to the ongoing enzymatic degradation of the C16-ApGltn molecules in the FS, as demonstrated in the *in vitro* degradation test (Fig. 3B). On Day 24, the blood flow in the C16-FS groups returned to an approximately normal level, and the C16-FS was considered to be almost completely degraded.

We also analyzed the difference in blood flow between groups at several time points (Fig. 4C). Although there was no statistically significant difference between the blood flows in the sham, Org-FS, and C16-FS groups on Day 1, C16-FS induced significantly higher blood flow on Day 3. Moreover, the C16-FS group sustained significantly higher blood flow up to Day 7 compared with the sham and Org-FS groups. However, the increased blood flow associated with C16-FS compared with Org-FS was no longer observed on Day 14. Comparing with the results of our previous study with an angiogenic self-assembled hydrogel,^27^ the C16-FS prepared with thermal crosslinking suppressed the subcutaneous spreading of material and successfully induced sustained localized angiogenesis for 14 days.

**Fig. 5:**
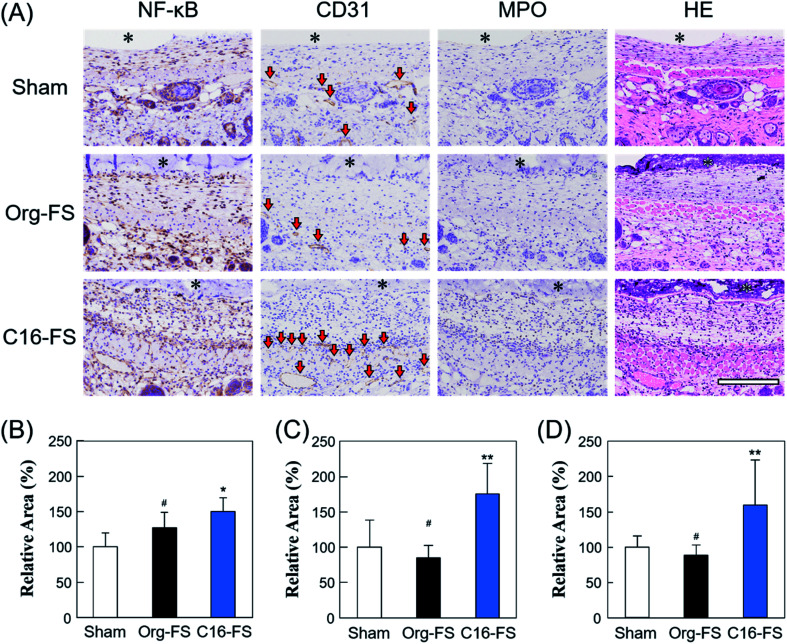
(A) Immunohistochemical studies of FSs and surrounding tissues 3 days after the sham operation and implantation of Org-FS or C16-FS (* denotes the FS, and the red arrows in the CD31-stained tissue sections indicate blood vessels). (B–D) Active areas of NF-kB, CD31, and MPO, respectively, as quantified using Image J (* indicates *p* < 0.05, ** indicates *p* < 0.01, and ^#^ indicates *p* > 0.05).

Fig. 5A shows tissue sections with immunohistochemical staining of NF-κB, CD31, MPO, and HE. The area of NF-κB staining revealed significantly increased NF-κB expression in the tissue around the C16-FS (Fig. 5A and B). NF-κB promotes genes associated with inflammatory cytokines and growth factors, such as VEGF, and we have previously shown that SFA-modified gelatin stimulates NF-κB *via* TLR4-mediated pathways.^27^ However, there were no statistically significant differences between the levels of VEGF staining in the Sham, Org-FS, and C16-FS implanted site, although the higher levels of VEGF staining was confirmed inside of C16-FS compared with Org-FS (Fig. S5†). We previously reported an increased affinity of VEGF to hydrophobically-modified gelatin based on surface plasmon resonance measurements.^51^ Therefore, VEGF was considered to be entrapped in the C16-FS by hydrophobic interactions between VEGF and the hexadecyl groups in C16-ApGltn, which results were correlated with the increased water contact angle of C16-FS. Furthermore, the level of the stained area of CD31, an endothelial marker, was elevated around the C16-FS implantation site compared with the sham and Org-FS (Fig. 5A and C). In addition, the number of blood vessels (indicated by red arrows in the CD31-stained sections) was higher in the C16-FS sample than in the Org-FS and sham samples, indicating that the C16-FS enhanced angiogenesis (Fig. 5A). The H&E staining showed that there was blood perfusion inside of the newly formed blood vessels around C16-FS (Fig. 5A). We further investigated the accumulation of granulocytes (including monocytes, neutrophils, and macrophages) by MPO staining, as shown in Fig. 5A and D. The observed increased accumulation of granulocytes around C16-FS was attributed to an increased foreign body reaction by the enhanced protein adsorption on C16-FS or a direct stimulation of inflammatory cells by hexadecyl groups.

In summary, C16-FS activated NF-κB secretion by stimulating TLR4-mediated pathways. NF-κB then activated the expression of VEGF, which became entrapped within the C16-FS and gradually released upon the degradation of C16-FS. The secreted VEGF promoted endothelial cell proliferation and, consequently, angiogenesis, which gave rise to increased blood perfusion (Fig. S5†). Based on these findings, it was concluded that C16-FS is a promising method to induce angiogenesis for prevascularization in cell or tissue transplantation without the addition of any growth factors. However, various types of inflammatory cytokines are secreted in response to VEGF, which is stimulated by NF-κB; the effects of those cytokines on implanted cells need to be further assessed in future studies. Moreover, long-term inflammation may lead to fibrosis,^52^ which would be a significant challenge for clinical application. Therefore, the incorporation of anti-inflammatory drugs may alleviate the inflammatory responses and modulate the balance of pro- and anti-inflammatory responses to lead the angiogenesis without fibrosis. The modification of cyclodextrin group in FSs enables not only the incorporation of anti-inflammatory drugs by forming inclusion complexes, but increased hydrophilicity, which prevents the adhesion of proteins on FSs *in vivo* followed by the foreign body reaction.^53^ Furthermore, we are now investigating how changing the thermal crosslinking time may influence the time-dependent modulation of the angiogenic response elicited by C16-FS.

## Conclusion

Here, we developed a C16-FS with thermal crosslinking to achieve sustained and localized angiogenesis *in vivo*. The C16-FS elicited a high water contact angle and exhibited a lower water absorption rate, indicating increased hydrophobicity of the C16-FS compared with the Org-FS. In addition, the C16-FS was stable in PBS for 24 h due to the thermal crosslinking, but the C16-FS was degraded by collagenase in 4 h (as was the Org-FS). *In vivo* experiments followed by LDPI observations revealed that the C16-FS induced significantly higher blood perfusion when implanted subcutaneously in mice compared with the Org-FS. Moreover, C16-FS induced sustained and localized angiogenesis for up to 7 days, followed by a decrease in blood flow to a normal level after 24 days. Immunohistochemical evaluations revealed that C16-FS promoted NF-κB secretion, leading to VEGF secretion, which was entrapped within the C16-FS and released, resulting in elevated CD31 expression and angiogenesis with blood perfusion. These results demonstrate that the C16-FS is a promising material for making vascular beads to improve the survivability of implanted cells or tissues without the need for drugs or growth factors.

## Data availability

The raw and processed data required to reproduce these findings cannot be shared at this time due to technical limitations.

## Conflicts of interest

The authors declare no conflict of interest.

## Supplementary Material

RA-010-D0RA03593A-s001
