# Supplementary material for: A hydrophobic gelatin fiber sheet promotes secretion of endogenous vascular endothelial growth factor and stimulates angiogenesis
Source: RSC Adv. 2020 Jun 30;10(42):24800–7. doi: 10.1039/d0ra03593a (PMC9055140; doi:10.1039/d0ra03593a)
Supplement: RA-010-D0RA03593A-s001 [file RA-010-D0RA03593A-s001.pdf]

Electronic Supplementary Material (ESI) for RSC Advances.
This journal is © The Royal Society of Chemistry 2020

A Hydrophobic Gelatin Fiber Sheet Promotes Secretion of Endogenous Vascular Endothelial Growth Factor and Stimulates Angiogenesis

Supporting Information

*Yosuke Mizuno[1] and Tetsushi Taguchi[1,2]**

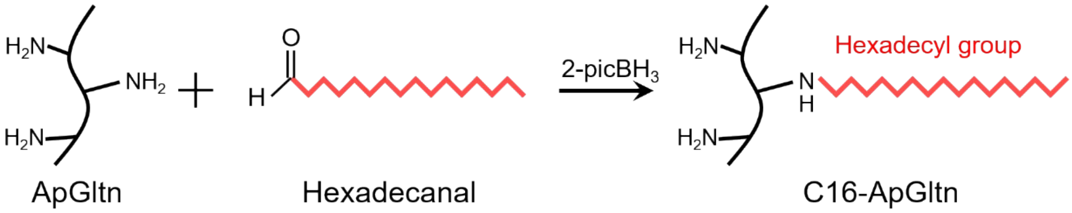

Figure S1. Synthesis of C16-ApGltn by reductive amination.

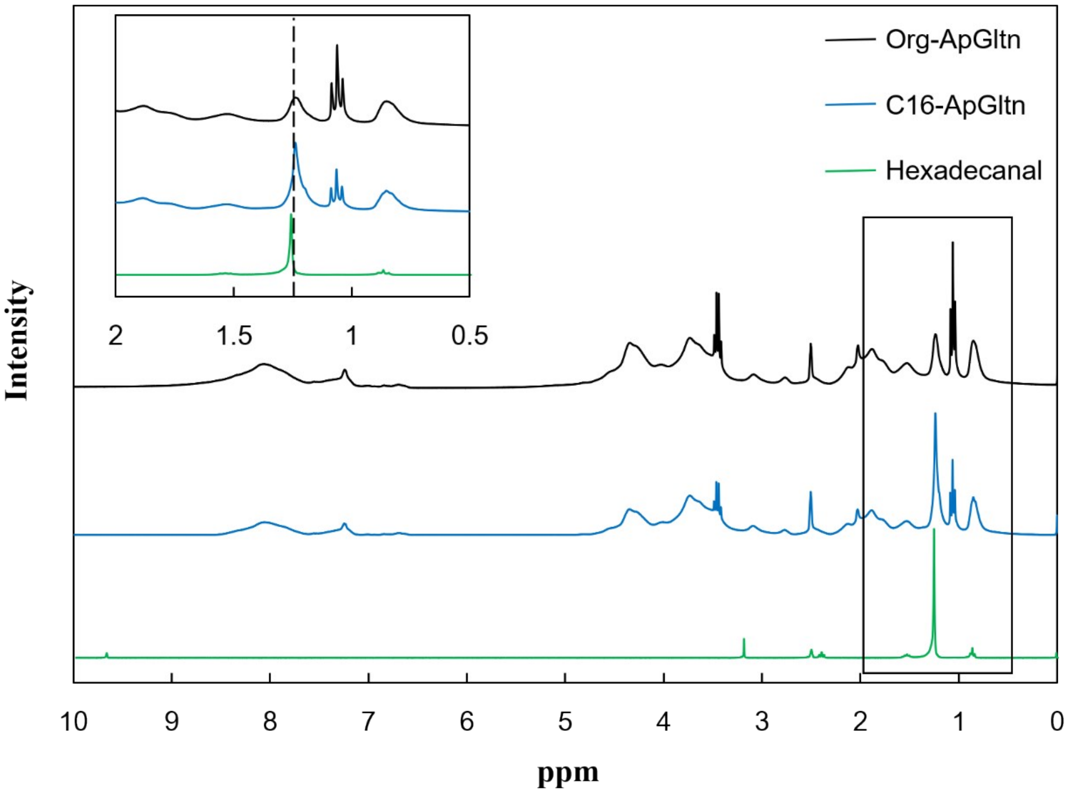

Figure S2. ¹H-NMR spectra of Org- and C16-ApGltn. The inset graph indicates the magnified spectra of the boxed region.

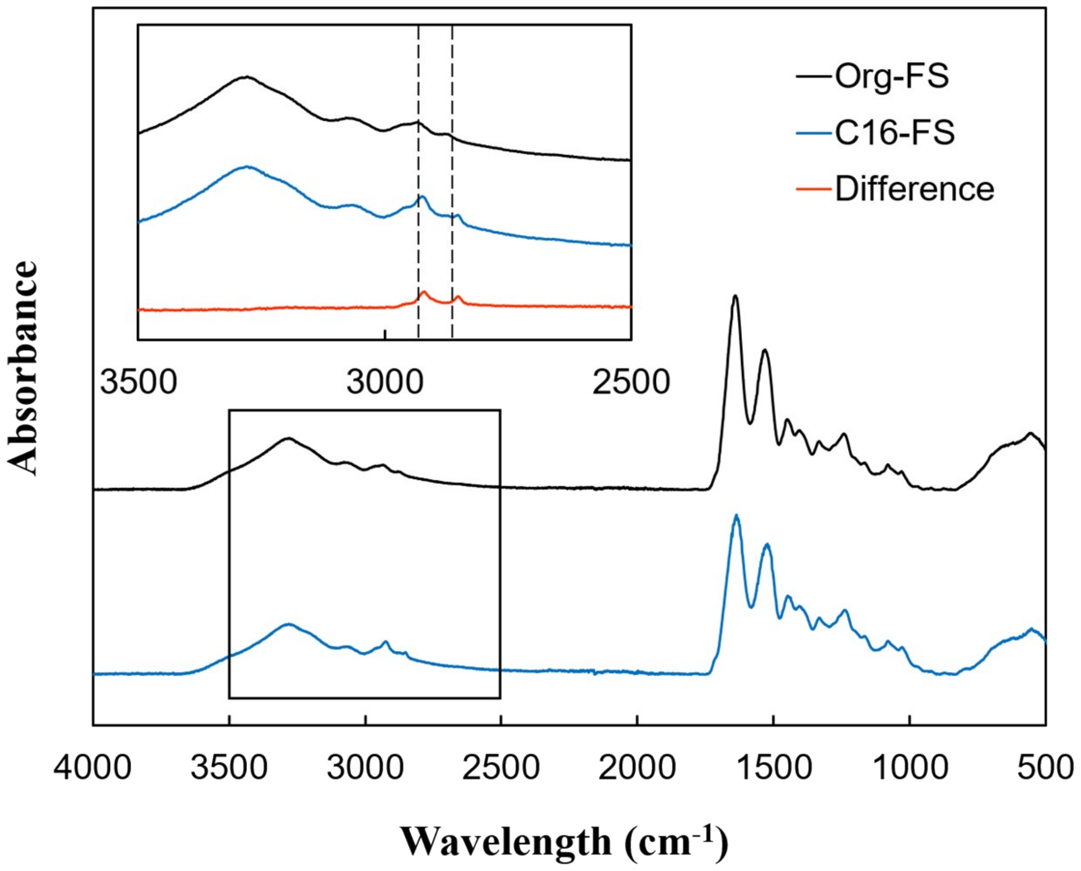

Figure S3. FT-IR spectra of Org- and C16-FS. (inset : magnified region in boxed area and the absorbance difference of Org- and C16-FS.)

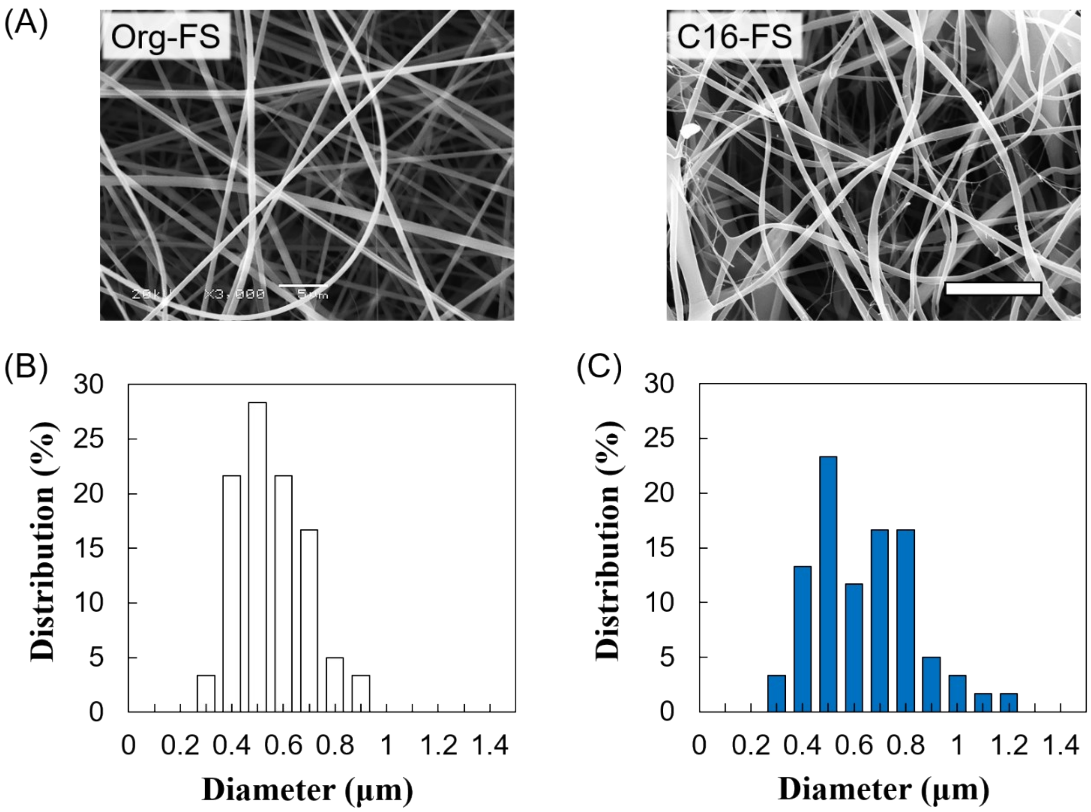

Figure S4. (A) SEM observation of Org- and C16-FS after the thermal crosslinking. (x3000) Scale bar = 5 μm. The fiber diameter histograms of (B) Org- and (C) C16-FS measured by Image J. (n=60).

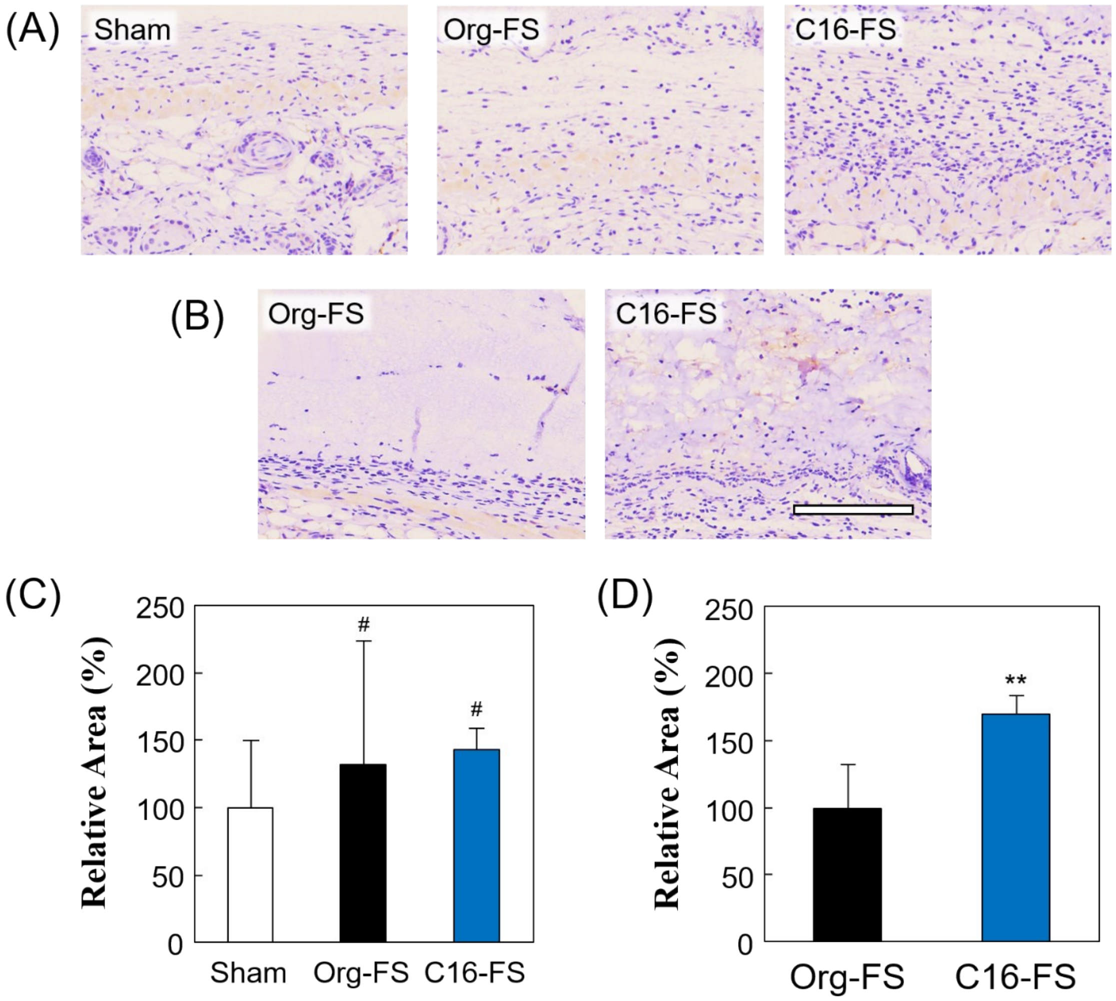

Figure S5. Evaluation of immunohistochemical expression of VEGF in (A) tissue and (B) FSs 3 days after the implantation of Org- and C16-FS. Scale bar = 200 μm. The VEGF expressed area in tissue and FSs was shown in (C) and (D), respectively. (n=10) $^{\#}p>0.05$, $^{**}p<0.01$.
